# Editorial: Future medical education in pediatrics and neonatology

**DOI:** 10.3389/fped.2023.1136323

**Published:** 2023-02-08

**Authors:** Michael Wagner, Philipp Deindl, Georg M. Schmölzer

**Affiliations:** ^1^ Division of Neonatology, Pediatric Intensive Care and Neuropediatrics, Department of Pediatrics, Comprehensive Center for Pediatrics, Medical University of Vienna, Vienna, Austria; ^2^ Department of Neonatology and Pediatric Intensive Care, Clinic for Gynecology and Obstetrics, University Hospital Hamburg Eppendorf, Hamburg, Germany; ^3^ Neonatal Research Unit, Centre for the Studies of Asphyxia and Resuscitation, Royal Alexandra Hospital, Edmonton, AL, Canada; ^4^ Department of Pediatrics, University of Alberta, Edmonton, AL, Canada

**Keywords:** simulation, education, virtual, training, neonatology

**Editorial on the Research Topic** Future medical education in pediatrics and neonatology

Pediatric and neonatal emergencies generate high-stress levels and an immense cognitive load for healthcare providers. For decades, a “see one, do one, teach one” approach was a common strategy within medical training (1). However, this approach is a challenge for patient safety, as providers used to perform procedures on patients for the very first time. Nowadays, a “see one, simulate many, do many, teach one” is acknowledged as more appropriate, where students and healthcare providers can practice skills and emergencies safely without harming patients (2). Simulation-based medical education is usually performed as either low-fidelity or high-fidelity training utilizing manikins and specific technology for on-site training (3). However, the COVID-19 pandemic demonstrated that traditional simulation-based medical education is not preserved from the outage and that healthcare and educational systems should be prepared for new educational challenges such as virtual teaching approaches (4). In this special issue of Frontiers in Pediatrics about future medical education in pediatrics and neonatology, we aimed to collect research articles focusing on promising and innovative new teaching methods for student training and clinical education.

## Virtual teaching

One strategy to overcome traditional training approaches with the need to be on-site, often limited due to staff shortages and lack of space to perform training, is a switch to virtual education strategies. Recently, there has been a significant increase in serious game applications (5). Serious games can augment learning and establish continuous algorithm and decision-making skills (5). The authors Bardelli et al. introduced a new computer game called “DIANA: Digital Application in Newborn Assessment”, which enables virtual training of neonatal life support on a computer, and the authors demonstrated the equivalence of this virtual training to conventional training. Furthermore, telemedicine for tele-simulation was also described as an option for distance training with the advantage of integrating external experts from other countries in the skill or team training process (6, 7). Löllgen et al. combined both serious gaming and tele-simulation utilizing avatars as surrogates for human participants to enable remote team training in multiple institutions simultaneously. They suggested this methodology as a feasible alternative to connect educators and trainees virtually at the same place. Whereas this training needs to be synchronized for participants, Wellmann et al. presented an asynchronous online training course with evidence-based content for neonatologists internationally.

However, future challenges will include the optimal integration and utilization of serious games and research on the outcome of virtual teaching methods on students’ and healthcare providers’ knowledge and preservation of psychological safety in a remote virtual setting.

## Individualized training

Future training approaches often utilize new technology or media (feedback devices, ultrasound, eye-tracking, augmented reality, video recording, 3D printing) compared to traditional training strategies or methods. These technologies are used and discussed for training and integration in clinical settings for real-time assessment (8). The utilization of video recording is an excellent example of how technology can be used to record simulations or real clinical situations for clinical education and research. After a critical or even only after a routine situation, a video recording, either with a designated video recording system or from a first-person perspective using eye-tracking glasses (9), can be reflected together with the whole team to identify problems such as the environment, the algorithm adherence, or teamwork and communication. After that, this knowledge can be used for targeted training to improve the workflow in the delivery room, intensive care unit, and individual and team behavior. Heesters et al. described in their article the integration of video recording and reflections in their local setting in combination with a narrative review about this technology for a change in team culture and an increase in patient safety. The article gives an excellent overview of necessary preconditions, technical issues, and the organization of video debriefings. While the optimal video recording system still needs to be determined, there are some advantages when using eye-tracking glasses, such as a first-person perspective as well as insights into the visual behavior of healthcare providers. This new technology has the potential to identify human factor issues and to learn more about individual behavior during routine and critical situations. Anesthetists have previously used this technology and identified visual attention’s influence on individual performance and workload (10). Gröpel et al. used eye-tracking in a cross-over randomized simulation trial and identified that a specific gaze behavior with a strong focus on the patient and a minimum of gaze transitions was correlated with improved outcomes of ventilations and chest compressions. Furthermore, this technology can be used for telemedicine, tele-simulation approaches, and generating new data in simulation-based medical education.

Besides video recording as a new educational tool, integrating objective feedback devices can play a significant role in training and supervision. Nowadays, most of the training is still performed using an instructor's subjective feedback. However, it has been shown that adding an objective feedback device, such as a respiratory function monitor, leads to better trainees' performance (11). Rod et al. confirmed that using a respiratory function monitor as objective feedback improved ventilation parameters. Moreover, real-time feedback in simulated and clinical situations can potentially decrease workload and improve patient outcomes. However, there are still many research questions about the optimal integration within a specific environment and the human-technology interaction before they can be recommended for routine use. Nevertheless, continuous data acquisition with feedback devices can help collect knowledge on individual performance.
